# A Phenolic-rich Extract of Cocoa (*Theobroma cacao* L.) Beans Impairs the Pathogenic Properties of *Porphyromonas gingivalis* and Attenuates the Activation of Nuclear Factor Kappa B in a Monocyte Model

**DOI:** 10.3389/froh.2022.867793

**Published:** 2022-03-22

**Authors:** Katy Vaillancourt, Amel Ben Lagha, Daniel Grenier

**Affiliations:** Oral Ecology Research Group, Faculty of Dentistry, Université Laval, Quebec, QC, Canada

**Keywords:** cocoa, epicatechin, oral keratinocytes, virulence factors, *Porphyromonas gingivalis*, periodontal disease

## Abstract

Periodontitis, an inflammatory disease that affects tooth-supporting tissues, is the result of a polymicrobial infection involving mainly Gram negative anaerobic bacteria. The aim of the present study was to investigate the effects of a phenolic-rich extract of cocoa (*Theobroma cacao* L.) beans on the pathogenic properties of *Porphyromonas gingivalis*, which is well-known as a keystone pathogen in the development of periodontitis. The effect of the cocoa extract on *P. gingivalis*-induced activation of the nuclear factor kappa B (NF-κB) transcription factor in a monocyte model was also assessed. The cocoa extract, whose major phenolic compound was epicatechin, inhibited the growth, hemolytic activity, proteolytic activities, and adherence properties (basement membrane matrix, erythrocytes) of *P. gingivalis* in a dose-dependent manner. It also protected the barrier function of a keratinocyte model against the deleterious effects mediated by *P. gingivalis*, and attenuated reactive oxygen species (ROS) production by oral keratinocytes treated with *P. gingivalis*. Lastly, the cocoa extract showed an anti-inflammatory property by preventing *P. gingivalis*-induced NF-κB activation in monocytes. In conclusion, this *in vitro* study highlighted the potential value of an epicatechin-rich extract of cocoa beans for preventing and/or treating periodontal diseases.

## Introduction

*Porphyromonas gingivalis*, an anaerobic Gram-negative bacterium, is known as a keystone pathogen in the development of periodontitis, which is a chronic inflammatory disease characterized by the progressive and irreversible destruction of the periodontal ligament and alveolar bone [1, 2]. The presence of *P. gingivalis* in subgingival biofilm is correlated with gingival bleeding, an increase in periodontal pocket depth, and bone resorption [1, 2]. This periodontal pathogen can express a large array of virulence factors, including adhesins, lipopolysaccharides (LPS), outer membrane vesicles, hemolysins, and proteinases [3–5]. These pathogenic determinants contribute to host colonization, nutrient acquisition, evasion of host immune defenses, and periodontal tissue destruction [3–5]. Arg- and Lys-gingipain cysteine proteinases, which are both extracellular and cell-bound, are the main endopeptidases produced by *P. gingivalis* [6, 7]. *P. gingivalis* gingipains have a broad spectrum of activity and degrade numerous host proteins such as type I collagen, fibronectin, and immunoglobulins [6, 7].

The oral mucosa is the first line of defense of the periodontium and acts as a barrier to protect the deeper periodontal connective tissues against bacterial invasion and toxin penetration [8, 9]. This protective function is provided by specialized intercellular tight junction proteins that seal the paracellular space between closely opposed cells and govern the permeability of the epithelial barrier [8, 9]. However, some periodontal pathogens such as *P. gingivalis* have evolved effective strategies to counteract the protective function of the epithelial barrier [10, 11]. Among others, the gingipains of *P. gingivalis* have been reported to damage cell-to-cell junctions and impair the epithelial barrier [10, 11].

*P. gingivalis* interactions with host cells through Toll-like receptors involving the nuclear factor kappa B (NF-κB) signaling pathway activate a complex inflammatory response that plays a crucial role in the progression and severity of periodontitis as well as associated systemic conditions [12, 13]. Macrophages and monocytes play a key role in host defenses against periodontal infections and contribute to the initiation of an adaptive immune response to periodontal pathogens such as *P. gingivalis* [14, 15]. These cells secrete many pro-inflammatory cytokines and matrix metalloproteinases (MMPs) [15, 16]. Although this host response contributes to gingival tissue homeostasis, excessive stimulation may result in the maintenance of a chronic inflammatory condition due to the continuous secretion of large amounts of inflammatory mediators involved in periodontal tissue destruction [12–15].

Current strategies for treating periodontitis include surgical intervention, mechanical therapy (ultrasonic debridement, scaling, and root planning), and the use of pharmacological agents [17, 18]. The objectives of these approaches are to clean periodontal pockets, polish root surfaces, and kill or eliminate bacterial biofilm. The pharmacological agents that exert an antibacterial effect or modulate the host response, enhance treatment outcomes [18, 19]. In this context, plant polyphenols have attracted the interest of several research groups due to their multi-target modes of action [20–22]. Cocoa (*Theobroma cacao* L.) is a plant containing a large variety of polyphenolic compounds [23, 24]. The beneficial effects of cocoa on human health have been mainly attributed to its high polyphenolic content. While studies have shown that cocoa polyphenols may be beneficial for treating cardiovascular diseases, endocrine disorders, and cancers, their benefits with regard to periodontal diseases have been poorly studied [25, 26].

Given that *P. gingivalis* is a major etiological agent of periodontitis, the aims of the present study were to investigate an epicatechin-rich cocoa extract for its ability to impair the pathogenic properties of *P. gingivalis* and attenuate *P. gingivalis*-induced activation of the NF-κB transcription factor in a monocyte model.

## Materials and Methods

### Cocoa Extract

A cocoa extract obtained by ethanol/water extraction of cocoa beans (*Theobroma cacao* L.) was obtained from Naturex (Avignon, France). A stock solution (20 mg/mL) was prepared in 10% dimethylsulfoxide (DMSO), sterilized by filtration (0.22 μm pore size), and kept at 4°C protected from light for up to 1 month. The phenolic characterization of the cocoa extract, as determined by chromatographic and mass spectrometry analyses, has been reported in a previous study [27]. The extract contains 30.93% (w/w) of polyphenols. Phenolic acids, flavonols, anthocyanins, flavan-3-ols, and procyanidins make up 0.15, 0.72, 0.12, 2.19, and 96.82% of the total polyphenols, respectively. Monomers, dimers, trimers, and tetramers make up 75% of the total procyanidin content.

### Bacteria and Growth Conditions

*P. gingivalis* ATCC 33277 was cultivated in Todd-Hewitt broth (THB; Becton Dickinson and Company, Sparks, MD, USA) supplemented with 0.001% (w/v) hemin and 0.0001% (w/v) vitamin K and was incubated in an anaerobic chamber (80% N_2_, 10% CO_2_, 10% H_2_) at 37°C.

### Minimum Inhibitory and Minimum Bactericidal Concentrations

A broth microdilution assay was used to determine the minimum inhibitory concentration (MIC) and minimum bactericidal concentration (MBC) values of the cocoa extract [28]. The MIC and MBC assays were performed in triplicate in three independent experiments.

### Hemagglutinating Activity

Erythrocytes were harvested from sheep blood (Nutri-Bact, Terrebonne, QC, Canada) by centrifugation (600 x *g* for 5 min), washed three times in 50 mM phosphate-buffered saline (pH 7.2, PBS), and suspended in the same buffer at a concentration of 2% (v/v). The effect of the cocoa extract on the hemagglutinating activity of *P. gingivalis* was assessed in a round-bottom microtiter plate. Two-fold serial dilutions of a *P. gingivalis* suspension (optical density at 660 nm [OD_660_] of 1.0 in PBS) were placed in the wells of the microplate in the absence or presence of the cocoa extract (3.9, 7.81, 15.63, 31.25 μg/mL). An equal volume (100 μL) of the erythrocyte suspension was added, and the microplate was shaken at room temperature for 30 min. After a further incubation at 4°C for 4 h, the reciprocal titer of the highest dilution of bacteria causing hemagglutination was recorded. The hemagglutination assay was performed in triplicate in three independent experiments.

### Hemolytic Activity

Equal volumes (1 mL) of the above erythrocyte suspension, *P. gingivalis* suspension, and two-fold serial dilutions of the cocoa extract (final concentrations in the assay: 3.9, 7.81, 15.625, 31.25, 62.5 μg/mL) were mixed and were incubated overnight at 37°C. PBS replaced the bacteria in the negative control. Following the incubation at 37°C, the mixtures were further incubated at 4°C for 1 h. They were then centrifuged (10,000 x *g* for 5 min), and the absorbance of the supernatants was read at 540 nm (A_540_). Hemolysis caused by *P. gingivalis* in the absence of the cocoa extract was assigned a value of 100%. Assays were performed in triplicate in three independent experiments, and the means ± standard deviations were calculated.

### Collagenase Activity

To determine the effect of the cocoa extract on the collagenase activity of *P. gingivalis*, a 48-h culture supernatant was incubated with the extract (final concentrations in the assay: 15.625, 31.25, 62.5, 125, 250 μg/mL) and the fluorescent substrate collagen DQ (Molecular Probes, Eugene, OR, USA) (100 μg/mL) in the wells of a clear bottom black wall microplate (Greiner Bio-One North America, Monroe, NC, USA) for 60 min at 37°C. The fluorescence corresponding to collagen degradation was then read using a Synergy 2 microplate reader (BioTek Instruments, Winooski, VT, USA), with the excitation and emission wavelengths set at 495 and 525 nm, respectively. The cocoa extract or the fluorescent substrate alone were used as controls. Leupeptin (100 μg/mL) was used as a positive inhibitor control. Collagenase activity obtained in the absence of the cocoa extract was assigned a value of 100%. Assays were performed in triplicate in three independent experiments, and the means ± standard deviations were calculated.

### Gingipain Activity

To evaluate the effect of the cocoa extract on the Arg- and Lys-gingipain activities of *P. gingivalis*, bacteria from an overnight culture were harvested by centrifugation, suspended in PBS (OD_660_ of 0.4), and added into the wells of a 96-well microplate. The bacteria were incubated at 37°C with 5 mM of either N-α-benzoyl-DL-arginine-*p*-nitroanilide (Arg-gingipain substrate) or N-*p*-tosyl-glycine-proline-lysine-*p*-nitroanilide (Lys-gingipain substrate) and 10 mM dithiothreitol in the presence or absence of the cocoa extract (15.625, 31.25, 62.5, 125, 250 μg/mL). Hydrolysis of the chromogenic substrates was monitored by reading the absorbance at 405 nm (A_405_) of the cell-free supernatant of the assay mixtures after 60 min (Arg-gingipain) or 18 h (Lys-gingipain) of incubation at 37°C. Tosyl-L-lysine chloromethyl ketone hydrochloride (4 mM) was used as a positive inhibitor control. Gingipain activity in the absence of the cocoa extract was assigned a value of 100%. Assays were performed in triplicate in three independent experiments, and the means ± standard deviations were calculated.

### Activity of Matrix Metalloproteinase 9

The effect of the cocoa extract (62.5, 125, 250 μg/mL) on MMP-9 activity was assessed using an MMP-9 Inhibitor Screening Fluorometric Assay Kit (Abcam Inc., Toronto, ON, Canada) according to the manufacturer's protocol. MMP-9 activity was monitored following a 20-min incubation at 37°C by recording fluorescence, with the excitation and emission wavelengths set at 328 nm and 420 nm, respectively. N-isobutyl-N-[4-methoxyphenylsulfonyl]glycyl hydroxamic acid (NNGH; 1 mM) was used as a control inhibitor of MMP-9 activity. MMP-9 activity in the absence of the cocoa extract was assigned a value of 100%. Assays were performed in triplicate in three independent experiments, and the means ± standard deviations were calculated.

### Adherence to a Basement Membrane Matrix Model

The effect of the cocoa extract on the adherence of *P. gingivalis* to Matrigel™ (BD Biosciences, Franklin Lakes, NJ, USA), a well-known *in vitro* basement membrane matrix model composed of major extracellular matrix proteins, including laminin, type IV collagen, and heparin sulfate proteoglycans, was assessed using fluorescein isothiocyanate (FITC)-labeled bacteria as described in a previous study [29]. Following a 2-h incubation of the bacteria with the Matrigel™ in the presence or absence of the cocoa extract (7.81, 15.625, 31.25, 62.5, 125, 250 μg/mL), relative fluorescence units (RUF; excitation wavelength 495 nm; emission wavelength 525 nm) corresponding to the level of bacterial adherence were read with a Synergy 2 microplate reader (BioTek Instruments). Wells with no bacteria were used as a control to determine basal autofluorescence, which was subtracted from the adherence values. Bacterial adherence in the absence of the cocoa extract was assigned a value of 100%. Assays were performed in triplicate in three independent experiments, and the means ± standard deviations were calculated.

### Transepithelial Electrical Resistance of Oral Keratinocytes

The human oral keratinocyte cell line B11 [30], which was kindly provided by S. Gröeger and J. Meyle (Justus-Lieig-University Giessen, Germany), was used to investigate the protective effect of the cocoa extract against *P. gingivalis*-mediated deleterious effects on keratinocyte barrier function. The cells were cultured in keratinocyte serum-free medium (K-SFM; Life Technologies Inc., Burlington, ON, Canada) supplemented with 50 μg/mL of bovine pituitary extract, 5 ng/mL of recombinant epidermal growth factor, and 100 μg/mL of penicillin G-streptomycin. Keratinocytes were seeded on Transwell^TM^ clear polyester membrane inserts (6.5-mm diameter, 0.4-μm pore size; Corning Co., Cambridge, MA, USA) at a concentration of 3 × 10^5^ cells per insert. The basolateral and apical chambers were filled with 0.6 mL and 0.1 mL of culture medium, respectively. After a 72-h incubation (37°C/5% CO_2_), the culture medium was replaced with fresh antibiotic-free K-SFM, and the cells were incubated for a further 16 h. Non-cytotoxic concentrations of the cocoa extract (31.25, 62.5, 125 μg/mL) identified in a previous study [27] were added to the apical chamber along with *P. gingivalis* at a multiplicity of infection (MOI) of 10^4^. The barrier function of the keratinocyte model was assessed by monitoring the transepithelial electrical resistance (TEER) at time 0 and after a 48-h incubation (37°C/5% CO_2_) using an ohmmeter (EVOM2, World Precision Instruments, Sarasota, FL, USA). Resistance values were calculated in Ohms (Ω)/cm^2^ by multiplying the resistance values by the surface area of the membrane filter. A 100% value was assigned to the TEER value at time 0. Assays were performed in triplicate in three independent experiments, and the means ± standard deviations were calculated.

### Reactive Oxygen Species Production by Oral Keratinocytes

A fluorometric assay was used to monitor the oxidation of 2', 7'-dichlorofluorescein-diacetate (DCF-DA; Sigma-Aldrich Canada Co., Oakville, ON, Canada) into a fluorescent compound that reflects reactive oxygen species (ROS) production. DCF-DA (40 mM) was freshly prepared in DMSO. B11 oral keratinocytes were seeded in the wells (10^5^ cells/well) of a 96-well microplate with black walls and a clear flat bottom (Greiner Bio-One North America) and were incubated overnight at 37°C in a 5% CO_2_ atmosphere. The cells were washed with Hank's balanced salt solution (HBSS; HyClone Laboratories, Logan, UT, USA) and were incubated for 30 min in the presence of 100 μM DCF-DA in HBSS. Excess DCF-DA was removed, and the keratinocytes were washed with HBSS. The cells were then treated with *P. gingivalis* (MOI of 10^3^ in HBSS) in the absence or presence of the cocoa extract (3.9, 7.8, 15.625, 31.25, 62.5, 125 μg/mL in culture medium). The fluorescence emission corresponding to ROS production was monitored after a 6-h incubation at 37°C using a Synergy 2 microplate reader (BioTek Instruments), with the excitation and emission wavelengths set at 485 nm and 528 nm, respectively. A 100% value was assigned to ROS production caused by *P. gingivalis* in the absence of the cocoa extract. Assays were performed in triplicate in three independent experiments, and the means ± standard deviations were calculated.

### NF-B Activation in a Monocyte Model

The human monoblastic leukemia cell line U937-3xκB-LUC, which was kindly provided by R. Blomhoff and H. Carlsen (University of Oslo, Norway), is a subclone of the U937 cell line stably transfected with a construct consisting of three NF-κB binding sites from the Ig κ light chain promoter coupled to the luciferase gene [31]. The cells were cultivated in Roswell Park Memorial Institute 1640 medium (RPMI-1640; Life Technologies Inc.) supplemented with 10% heat-inactivated fetal bovine serum (FBS), 100 μg/mL of penicillin/streptomycin, and 75 μg/mL of hygromycin B at 37°C in a 5% CO_2_ atmosphere. The effect of the cocoa extract (3.9, 7.81, 15.625, 31.25, 62.5, 125 μg/mL) on *P. gingivalis*-induced NF-κB activation was assessed. These concentrations were found to be non-cytotoxic in a previous study [27]. The monocyte suspension (50 μL, 3 × 10^6^ cells/mL) was seeded in the wells of a black bottom, black wall 96-well microplate (Greiner Bio-One North America). The cocoa extract (50 μL) was added and the microplate was incubated for 30 min at 37°C in a 5% CO_2_ atmosphere. *P. gingivalis* (50 μL, MOI of 10^2^) was then added to induce the activation of the NF-κB signaling pathway, and the plate was incubated for a further 6 h at 37°C in a 5% CO_2_ atmosphere. NF-κB activation was then monitored using a commercial luciferase assay kit (Bright-Glo™ Luciferase Assay System; Promega, Madison, WI, USA) by adding 100 μL of the luciferase substrate to each well. Luminescence was recorded using the luminometer option of a Synergy 2 microplate reader (BioTek Instruments). BAY 11-7082 (Selleckchem, Houston, TX, USA), a known inhibitor of the NF-κB pathway, was used as a positive control. A 100% value was assigned to the NF-κB activation caused by *P. gingivalis* in the absence of cocoa extract. Assays were performed in triplicate in three independent experiments, and the means ± standard deviations were calculated.

### Statistical Analysis

A one-way ANOVA with a *post-hoc* Bonferroni multiple comparison test was used to analyze the data. Results were considered statistically significant at *p* < 0.01 or *p* < 0.001.

## Results

### Effects on the Growth and Virulence Properties of *P. Gingivalis*

**Figure 1:**
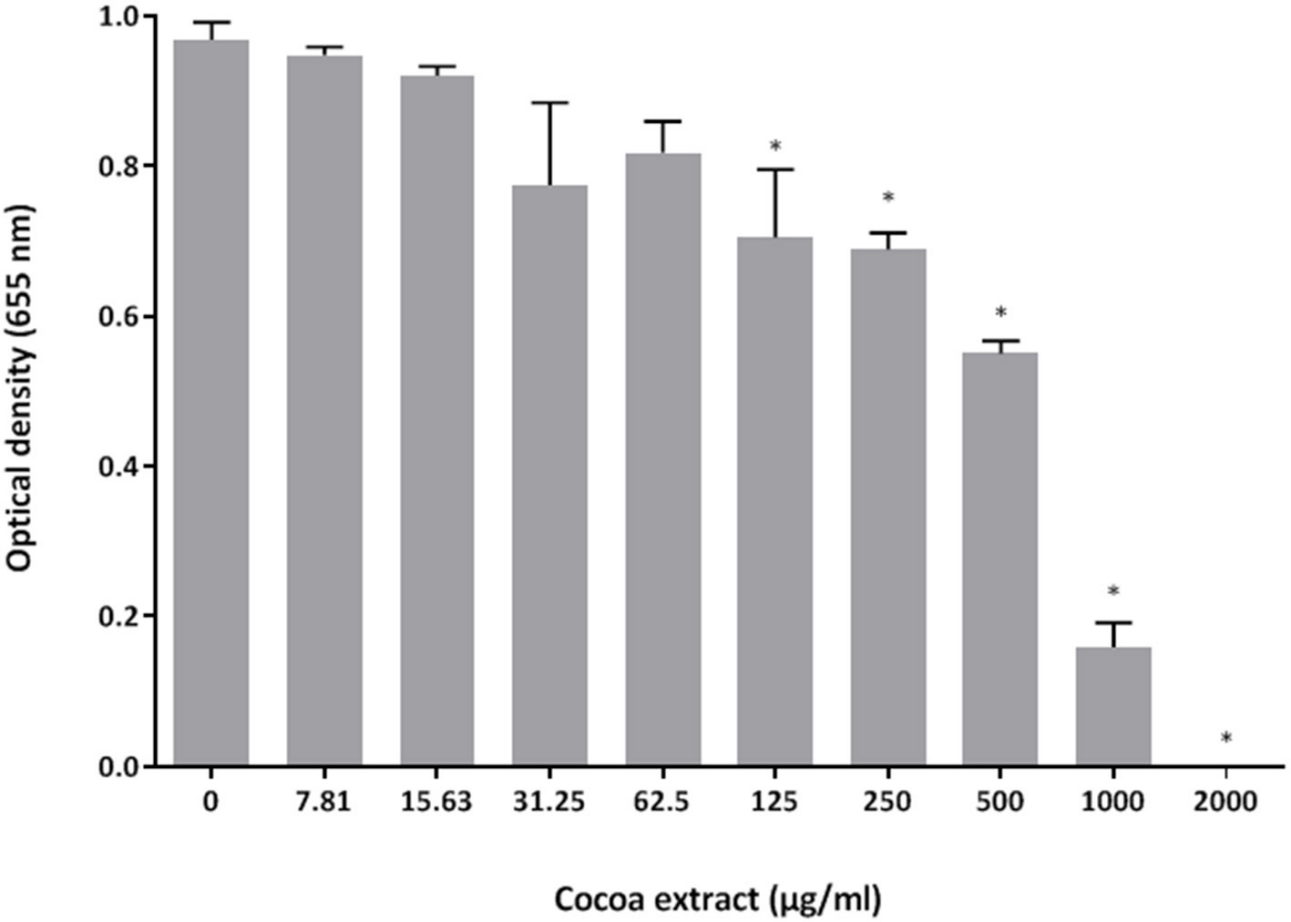
Effect of the cocoa extract on the growth of *P. gingivalis*. Bacterial growth was monitored by recording the optical density at 655 nm following a 24-h incubation at 37°C under anaerobiosis. *significant inhibition (*p* < 0.01) compared to the control (no cocoa extract).

The antibacterial activity of the epicatechin-rich cocoa extract against the reference strain *P. gingivalis* ATCC 33277 was assessed using a broth microdilution assay. As shown in Figure 1, the extract dose-dependently inhibited the growth of *P. gingivalis*. A concentration of 1,000 μg/mL reduced bacterial growth by 84% while 2,000 μg/mL completely inhibited growth. No MBC value was obtained.

**Figure 2:**
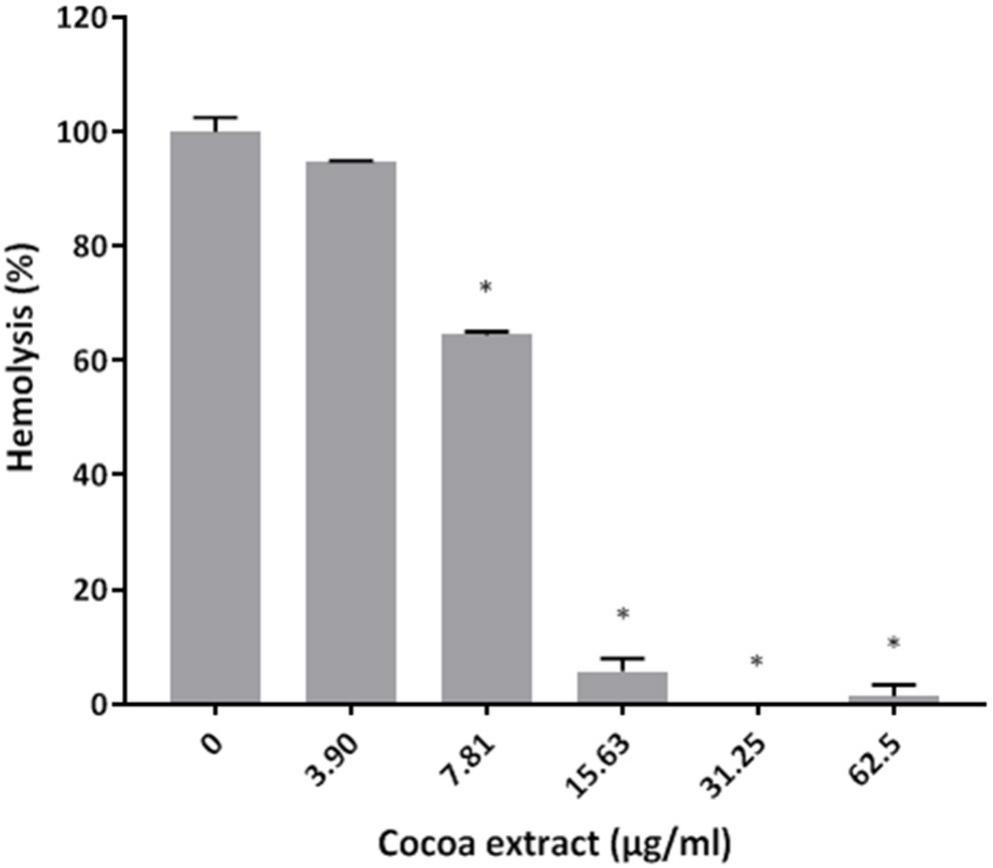
Effect of the cocoa extract on the hemolytic activity of *P. gingivalis* cells. Hemolysis of sheep red blood cells was monitored by recording the absorbance at 540 nm (A_540_) following an overnight incubation at 37°C and a one-h incubation at 4°C. A relative value of 100% was assigned to hemolysis obtained in the absence of the cocoa extract. *significant inhibition (*p* < 0.01) compared to the control (no cocoa extract).

The effects of the epicatechin-rich cocoa extract on selected virulence properties of *P. gingivalis* were then investigated. In a hemolytic assay using sheep erythrocytes, the extract dose-dependently inhibited hemolysis (Figure 2). Complete inhibition was obtained with cocoa extract concentrations as low as 31.25 μg/mL.

**Figure 3:**
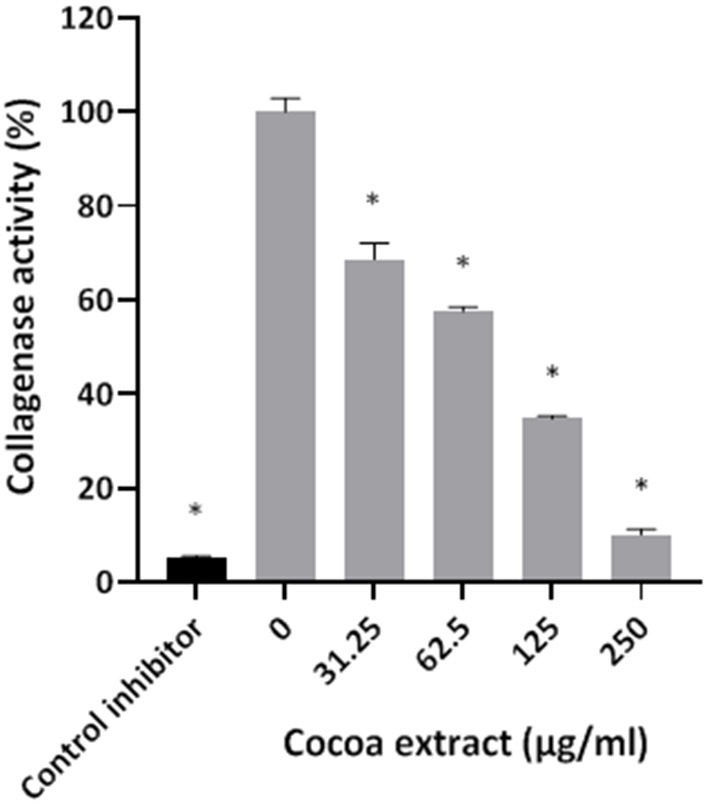
Effect of the cocoa extract on the collagenase activity of *P. gingivalis*. Leupeptin was used as a control inhibitor. Collagen degradation was monitored by recording the fluorescence at excitation and emission wavelengths of 495 and 525 nm, respectively, following a 60-min incubation at 37°C. A relative value of 100% was assigned to the degradation of type I collagen obtained in the absence of the cocoa extract. *significant inhibition (*p* < 0.01) compared to the control (no cocoa extract).

**Figure 4:**
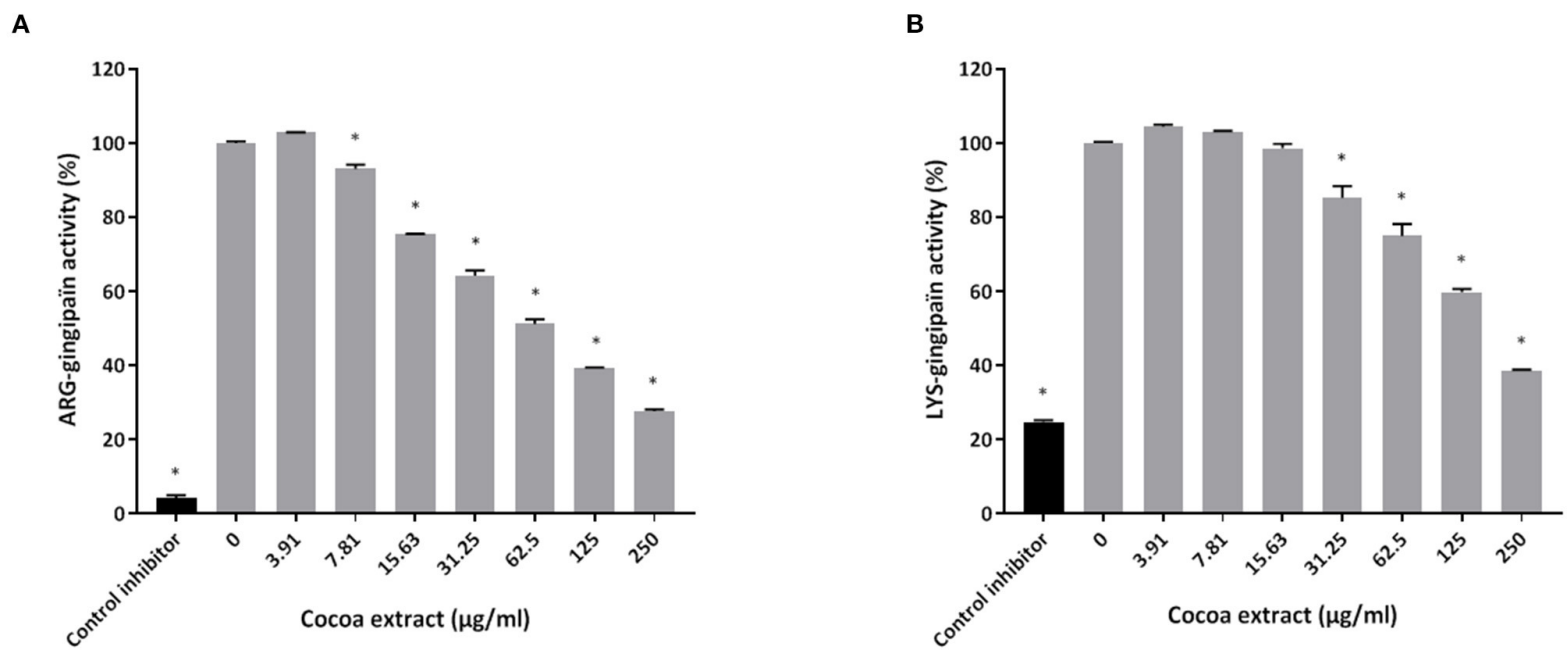
Effect of the cocoa extract on the Arg-gingipain activity **(A)** and Lys-gingipain activity **(B)** of *P. gingivalis*. Tosyl-L-lysine chloromethyl ketone hydrochloride was used as a control inhibitor. Hydrolysis of the chromogenic substrates was monitored by recording the absorbance at 405 nm (A_405_) after 60 min (Arg-gingipain) or 18 h (Lys-gingipain) of incubation at 37°C. A relative value of 100% was assigned to the activity obtained in the absence of the cocoa extract. *significant inhibition (*p* < 0.01) compared to the control (no cocoa extract).

**Figure 5:**
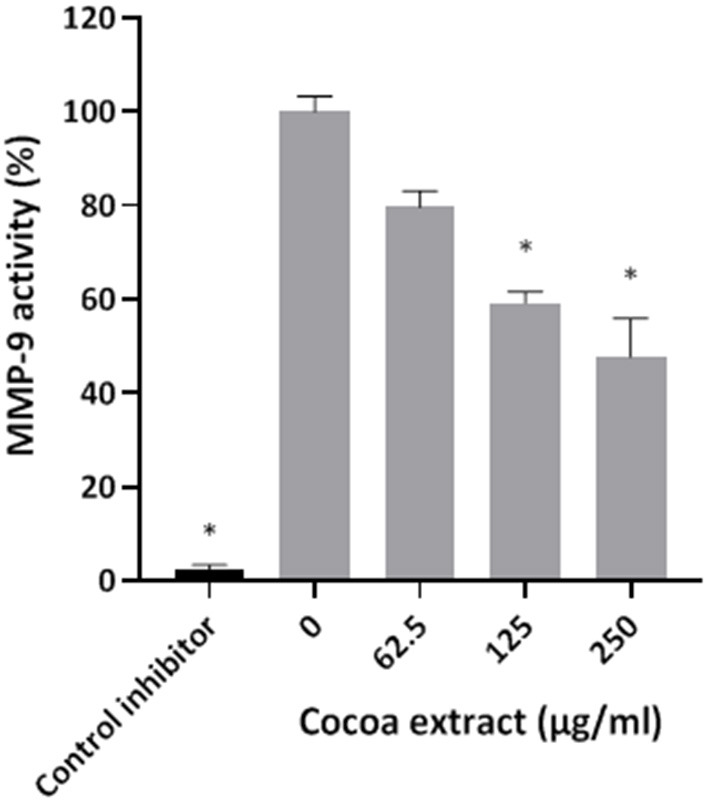
Effect of the cocoa extract on MMP-9 activity. N-isobutyl-N-[4-methoxyphenylsulfonyl]glycyl hydroxamic acid was used as a control inhibitor. MMP-9 activity was monitored by recording the fluorescence at excitation and emission wavelengths of 328 and 420 nm, respectively, following a 20-min incubation at 37°C. A relative value of 100% was assigned to the activity obtained in the absence of the cocoa extract. *significant inhibition (*p* < 0.01) compared to the control (no cocoa extract).

Thereafter, the ability of the cocoa extract to reduce the degradation of type I collagen by proteinases in a culture supernatant of *P. gingivalis* was investigated. A significant dose-dependent inhibition was observed. More specifically, 250 μg/mL of the extract reduced the *P. gingivalis*-mediated degradation of type I collagen by 89.9%, while a concentration of 31.25 μg/mL reduced degradation by 31.4% (Figure 3). We also tested the effect of the epicatechin-rich cocoa extract on the cell-associated Arg- and Lys-gingipain activities of *P. gingivalis*. The extract dose-dependently decreased the activity of Arg-gingipain (Figure 4A) and, to a lesser extent, Lys-gingipain (Figure 4B). At a concentration of 250 μg/mL, the cocoa extract reduced Arg-gingipain activity by 72.3% and Lys-gingipain activity by 61.3%. Based on this interesting observation that the epicatechin-rich cocoa extract inhibits bacterial proteolytic enzymes, we hypothesized that it may also be effective against host-derived MMPs that are known to contribute to tissue destruction during periodontitis. We used MMP-9 as a model and found that the cocoa extract inhibits the catalytic activity of MMP-9 in a dose-dependent manner (Figure 5) with 62.5, 125, and 250 μg/mL of the cocoa extract inhibiting MMP-9 activity by 20.4, 40.9, and 52.3%, respectively.

**Figure 6:**
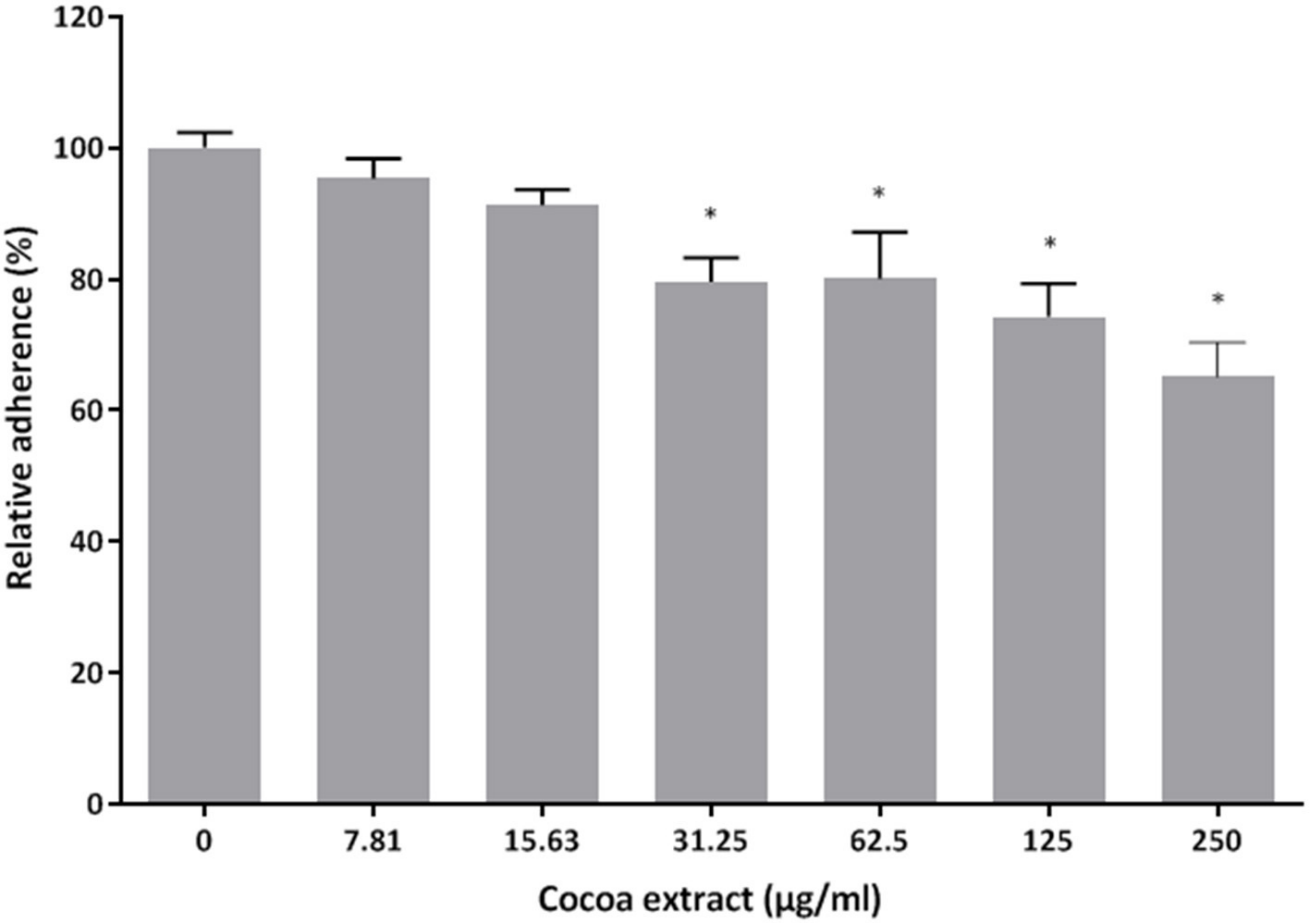
Effect of the cocoa extract on the adherence of *P. gingivalis* to the Matrigel™ basement membrane model. Adherence of FITC-labeled bacteria was monitored by recording the fluorescence at excitation and emission wavelengths of 495 and 525 nm, respectively, following a 2-h incubation. A relative value of 100% was assigned to the adherence obtained in the absence of the cocoa extract. *significant inhibition (*p* < 0.01) compared to the control (no cocoa extract).

The ability of the cocoa extract to inhibit the adherence of *P. gingivalis* was then evaluated. Untreated *P. gingivalis* cells had an hemagglutination titer of 8 in a sheep erythrocyte assay (Table 1). The titer decreased to 2 in the presence of 3.9 μg/mL of the cocoa extract. Surprisingly, ≥ 15.625 μg/mL of the cocoa extract promoted the agglutination of erythrocytes, even in the absence of *P. gingivalis*. The effect of the epicatechin-rich cocoa extract on the adherence of *P. gingivalis* to a polystyrene surface coated with Matrigel™, a basement membrane matrix model, was also assessed. At the highest concentration tested (250 μg/mL), the cocoa extract inhibited adherence to the Matrigel™-coated polystyrene surface by 34.9% (Figure 6).

**Table 1:** Effect of the cocoa extract on the hemagglutinating activity of *P. gingivalis*.

**Cocoa extract (μg/mL)**	Hemagglutination titer ^*^
0	8
3.9	2
7.81	4
15.63	16
31.25	32

* *Reciprocal of the highest dilution of the bacterial suspension that produced complete agglutination of the sheep erythrocytes*.

### Effect on *P. Gingivalis*-Induced Loss of Oral Keratinocyte Barrier Function

**Figure 7:**
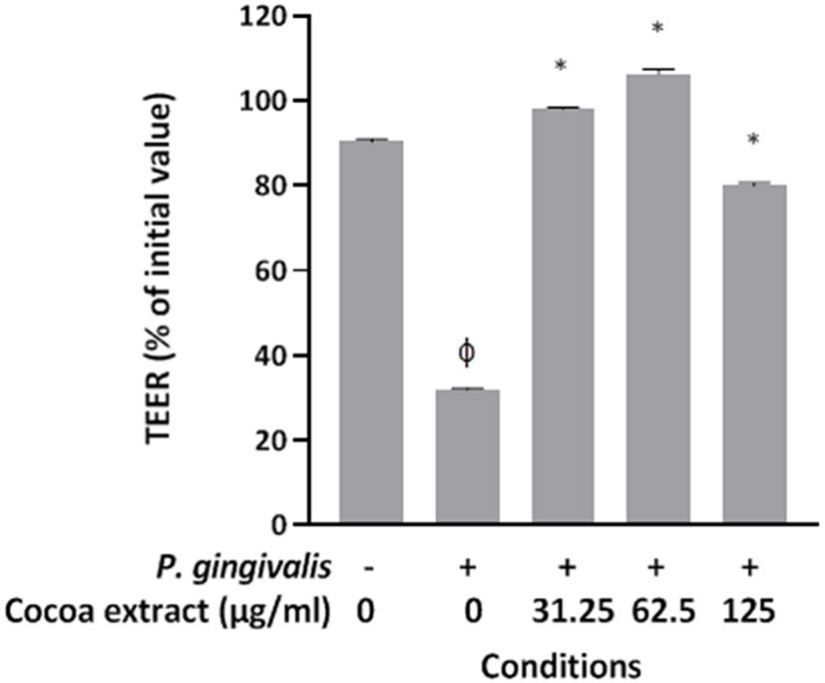
Effect of the cocoa extract on *P. gingivalis*-mediated loss of oral keratinocyte barrier function. Bacteria were used at MOI of 10^4^, and the barrier function of the keratinocyte model was assessed by monitoring the transepithelial electrical resistance (TEER) values at time 0 and after a 48-h incubation (37°C/5% CO_2_) using an ohmmeter. A relative value of 100% was assigned to the TEER recorded at time 0. Results are expressed as the means ± SD of triplicate assays from three independent experiments. φ, significant decrease (*p* < 0.001) compared to unstimulated control cells. *, significant increase (*p* < 0.001) compared to *P. gingivalis*-stimulated cells not treated with the cocoa extract.

Given that *P. gingivalis* has been reported to have deleterious effects on keratinocyte barrier integrity [10, 11], we determined whether the epicatechin-rich cocoa extract protects oral keratinocytes from this damage. Treating the keratinocytes for 48 h with *P. gingivalis* at an MOI of 10^4^ significantly decreased TEER by 68.1% compared to control untreated cells (Figure 7). However, the presence of the cocoa extract at all the concentrations tested (31.25, 62.5, 125 μg/mL) significantly reduced the *P. gingivalis*-induced decrease in TEER in the oral keratinocyte model used.

### Effect on *P. Gingivalis*-Induced ROS Production by Oral Keratinocytes

**Figure 8:**
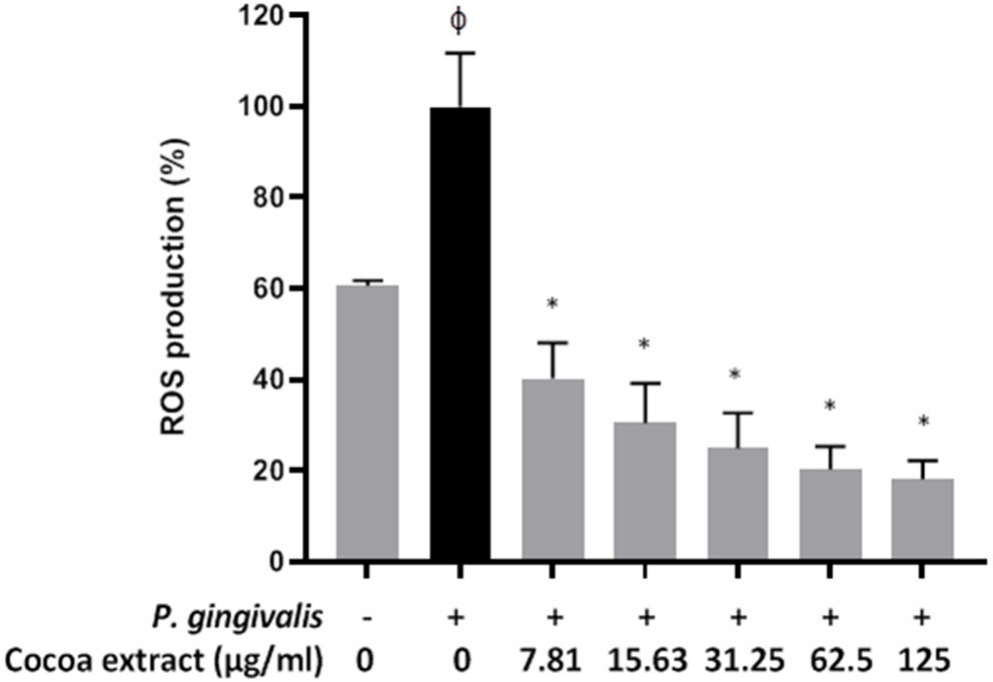
Effect of the cocoa extract on *P. gingivalis*-induced ROS production by oral keratinocytes. Bacteria were used at MOI of 10^3^, and ROS production was monitored by recording the fluorescence at excitation and emission wavelengths of 485 and 528 nm, respectively, following a 6-h incubation (37°C/5% CO_2_). A relative value of 100% was assigned to ROS production in the absence of the cocoa extract. φ, significant increase compared to the control (no *P. gingivalis* stimulation). *significant inhibition (*p* < 0.001) compared to the control (no cocoa extract + *P. gingivalis* stimulation).

Treating the oral keratinocytes with *P. gingivalis* (6 h) increased the production of ROS 1.64-fold compared to untreated control cells (Figure 8). However, the presence of the cocoa extract at all the concentrations tested (7.81 to 125 μg/ml) significantly reduced *P. gingivalis*-induced ROS production (Figure 8). ROS levels decreased by 61.4% with 7.81 μg/mL of cocoa extract.

### Effect on *P. Gingivalis*-Induced NF-κB Activation in a Monocyte Model

**Figure 9:**
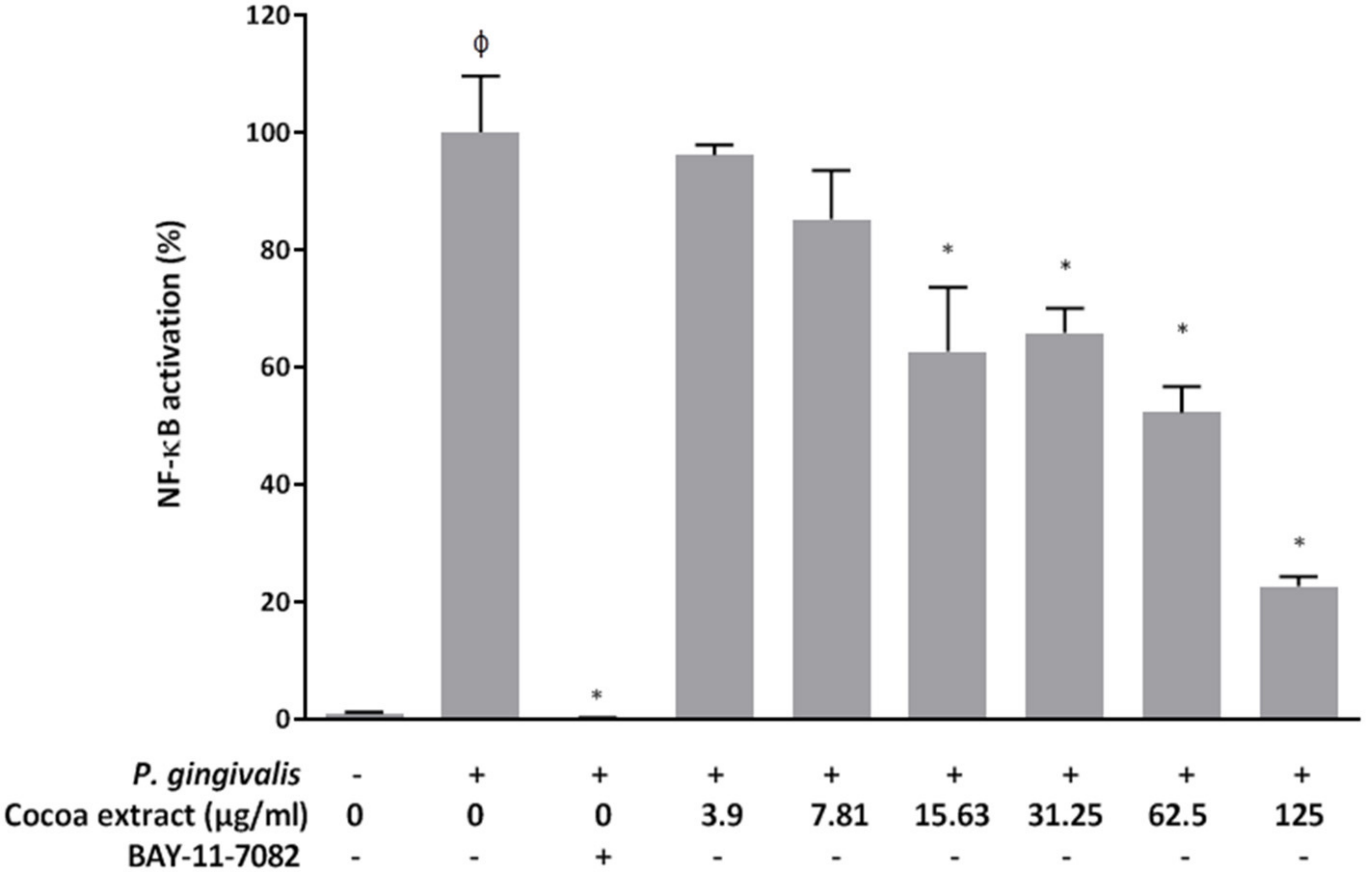
Effect of the cocoa extract on the activation of the NF-κB signaling pathway induced by *P. gingivalis* (MOI of 10^2^) using the human monoblastic leukemia cell line U937-3xκB-LUC. BAY-11-7082 was used as a control inhibitor. NF-κB activation was monitored by recording luminescence after a 6-h incubation (37°C/5% CO_2_). A value of 100% was assigned to the activation in the absence of the cocoa extract. φ, significant increase (*p* < 0.001) compared to the control (no *P. gingivalis* stimulation). *significant inhibition (*p* < 0.001) compared to the control (no cocoa extract + *P. gingivalis* stimulation).

To assess the anti-inflammatory potential of the epicatechin-rich cocoa extract, we examined its ability to reduce *P. gingivalis*-induced NF-κB activation using the U937-3xκB monocytic cell line. As shown in Figure 9, the cocoa extract dose-dependently reduced *P. gingivalis*-induced NF-κB activation. Significant inhibitory effects (34.2% to 77.4%) were observed at concentrations of the cocoa extract ranging from 15.625 to 125 μg/mL. As expected, the commercial positive inhibitory control (BAY-11-7082) caused a complete inhibition of NF-κB activation.

## Discussion

Increasing evidence suggests that the phytochemical constituents of cocoa, particularly polyphenols, demonstrate beneficial health properties with the capacity to improve and prevent several human disorders [24–26]. However, the potential benefits of cocoa in terms of periodontal health have been poorly studied to date. In the present study, we hypothesized that an epicatechin-rich cocoa extract may impair the growth and virulence properties of *P. gingivalis* and attenuate the activation of the NF-κB transcription factor induced by this periodontal pathogen in a monocyte model.

We first showed that the cocoa extract completely inhibits the growth of *P. gingivalis* when added to the culture medium at a concentration of 2,000 μg/mL. Since no MBC value was obtained, it suggests that the cocoa extract has a bacteriostatic mode of action, although a bactericidal effect may be obtained at concentrations >2,000 μg/mL. Given that the cocoa extract contains a variety of polyphenols [27], it is likely that the antibacterial mechanism of action against *P. gingivalis* involves different targets. However, the growth inhibitory effect may be related to the ability of epicatechin, which is the main phenolic compound in the cocoa extract, to bind to the lipid bilayer of the bacterial membrane, resulting in the disruption of the cell structure, leading to cell death [32]. Previous studies have also reported that cocoa extracts exert antibacterial activity against periodontal pathogens, including *Fusobacterium nucleatum, Prevotella intermedia*, and *P. gingivalis* [27, 33].

The ability of *P. gingivalis* to cause lysis of erythrocytes and release hemoglobin is considered a virulence determinant since it provides an iron source to periodontal pathogens that promotes their proliferation in periodontal pockets [34]. Interestingly, the cocoa extract inhibited the hemolytic activity of *P. gingivalis* in a dose-dependent manner. By reducing the release of hemoglobin, the cocoa extract may not only impair the proliferation of *P. gingivalis* but may also reduce the levels of pro-inflammatory mediators in periodontal sites. Bodet et al. [35] previously reported that hemoglobin can synergize with *P. gingivalis* LPS to amplify the inflammatory response of human macrophages.

*P. gingivalis* produces several proteolytic enzymes active on a variety of host proteins thus contributing to local tissue destruction and immune defense perturbation [6, 7]. Inhibiting these enzymes may be an effective therapeutic strategy to prevent the initiation and progression of periodontal disease. In the present study, the cocoa extract attenuated collagen degradation by *P. gingivalis*. It also inhibited cell surface Arg- and Lys-gingipains, which are the main endoproteinases produced by *P. gingivalis*. Given this anti-proteinase property of the cocoa extract, we further investigated this aspect by assessing its effect on the activity of host MMPs, which are major contributors to periodontal tissue destruction [36, 37]. MMP-9 was used as model as it is found in high amounts in inflamed periodontitis sites and is strongly associated with the progression and severity of periodontitis [36–38]. The present study showed that the cocoa extract exerts a marked dose-dependent inhibitory effect on the catalytic activity of MMP-9, suggesting that it may help reduce periodontal tissue destruction.

The adhesion of *P. gingivalis* to matrix constituents of the basement membrane is crucial for the colonization of periodontal sites by this periodontal pathogen [3–5]. In the present study, we showed that the cocoa extract can impair the adherence of *P. gingivalis* to Matrigel™, a well-known basement membrane model containing laminin and type IV collagen as the main protein constituents. As we observed that the cocoa extract also reduces the hemagglutinating activity of the bacterium, this effect likely occurs due to the neutralization of hemagglutinins, which are key adhesins expressed by *P. gingivalis* [39].

The oral mucosal epithelium is a selective protective barrier that prevents the translocation of periodontal pathogens into gingival connective tissues [8, 9]. In a previous investigation, we showed that the cocoa extract used in the present study can improve the barrier function of an oral keratinocyte model by increasing TEER. As the gingipains produced by *P. gingivalis* can destroy cell-to-cell junctions, resulting in the disruption of epithelial barrier function [10, 11], we determined whether the cocoa extract protects against the *P. gingivalis*-mediated deleterious effects. We showed that the epicatechin-rich cocoa extract can protect the integrity of the oral keratinocyte barrier by preventing the decrease in TEER despite a 48-h challenge with *P*. *gingivalis*. Part of this protective effect may be related to the ability of the cocoa extract to inhibit *P. gingivalis* gingipains.

ROS production by mucosal and immune cells significantly increases during chronic periodontal inflammations and results in oxidative stress [40, 41]. Excessive amounts of ROS may damage DNA, proteins, and lipids, leading to cell death. We showed that *P. gingivalis* induces the production of intracellular ROS in the oral keratinocyte model used and that the addition of the cocoa extract during keratinocyte stimulation significantly inhibits ROS production in a dose-dependent manner.

The transcription factor NF-κB plays a central role in inflammatory disorders such as periodontitis by promoting the expression of a large array of genes encoding pro-inflammatory cytokines [42]. The inhibition of the NF-κB signaling pathway has thus been proposed as a promising therapeutic strategy [43]. In the present study, we showed that the cocoa extract can attenuate *P. gingivalis*-induced NF-κB activation in a monocyte model, which is in agreement with our previous study using another periodontal pathogen (*F. nucleatum*) [27]. This anti-inflammatory property may involve the ability of epicatechin, catechin, and procyanidins in *T. cacao* to modulate NF-κB binding to DNA [44–46]. Additional *in vitro* models, including gingival mesenchymal stem cells and periodontal ligament stem cells that has been used to demonstrate the ability of ascorbic acid to down-regulate the inflammatory cascade induced by *P. gingivalis* LPS [47, 48], should be tested to confirm the anti-inflammatory property of the cocoa extract.

In summary, the present study provided evidence that the epicatechin-rich cocoa extract under investigation impairs various pathogenic properties of *P. gingivalis*, a keystone pathogen in periodontitis. Moreover, the cocoa extract attenuated the inflammatory process associated with periodontitis by inhibiting the NF-κB signaling pathway. Interestingly, Tomofuji et al. [49] reported that a cocoa-enriched diet could reduce alveolar bone loss as well as leukocyte infiltration in a rat-periodontitis model. Further research is required to evaluate the clinical efficacy of cocoa polyphenols incorporated into mouthrinses, toothpastes, gels, or local drug delivery systems.

## Data Availability Statement

The original contributions presented in the study are included in the article/supplementary material, further inquiries can be directed to the corresponding author.

## Author Contributions

DG conceived and designed the experiments and wrote the manuscript. KV and AB performed the experimental assays and the statistical analyses. All authors approved the submitted version.

## Funding

This study was supported by the Fonds Émile-Beaulieu.

## Conflict of Interest

The authors declare that the research was conducted in the absence of any commercial or financial relationships that could be construed as a potential conflictof interest.

## Publisher's Note

All claims expressed in this article are solely those of the authors and do not necessarily represent those of their affiliated organizations, or those of the publisher, the editors and the reviewers. Any product that may be evaluated in this article, or claim that may be made by its manufacturer, is not guaranteed or endorsed by the publisher.
